# Supplementary material for: Effects of the Interface Properties on the Performance of UV-C Photoresistors: Gallium Oxide as Case Study
Source: Sensors (Basel). 2025 Jan 9;25(2):345. doi: 10.3390/s25020345 (PMC11769345; doi:10.3390/s25020345)
Supplement: Supplementary file 1 [file sensors-25-00345-s001.zip › sensors-3341079-supplementary.pdf]

sensors

MDPI

Supporting Information

Effects of the Interface Properties on the Performance of UV-C Photoresistors: Gallium Oxide as Case Study

Maura Pavesi [1,*], Antonella Parisini [1], Pietro Calvi [1], Alessio Bosio [1], and Roberto Fornari [1,2]

[1] Department of Mathematical, Physical and Computer Sciences, University of Parma, Viale delle Scienze 7/A, 43124 Parma, Italy; antonella.parisini@unipr.it (A.P.); pietro.calvi@unipr.it (P.C.); alessio.bosio@unipr.it (A.B.); roberto.fornari1@unipr.it (R.F.)
[2] IMEM-CNR Institute, Viale delle Scienze 37/A, 43124 Parma, Italy
* Correspondence: maura.pavesi@unipr.it

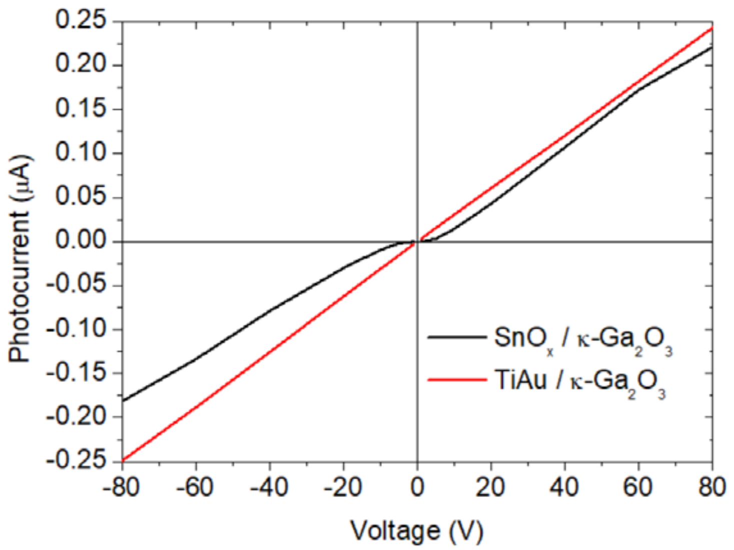

Figure S1. Comparison between the I-V curves of photoresistors with SnO_x and Ti/Au contacts deposited on the same Ga_2O_3 layer measured on the pair of contacts 1–2 (200 µm). The sample with Ti/Au contacts shows a very good linearity over a wide range of applied voltage. The photoresistor with SnO_x contacts shows, instead, the effect of the contact resistance at low voltages.

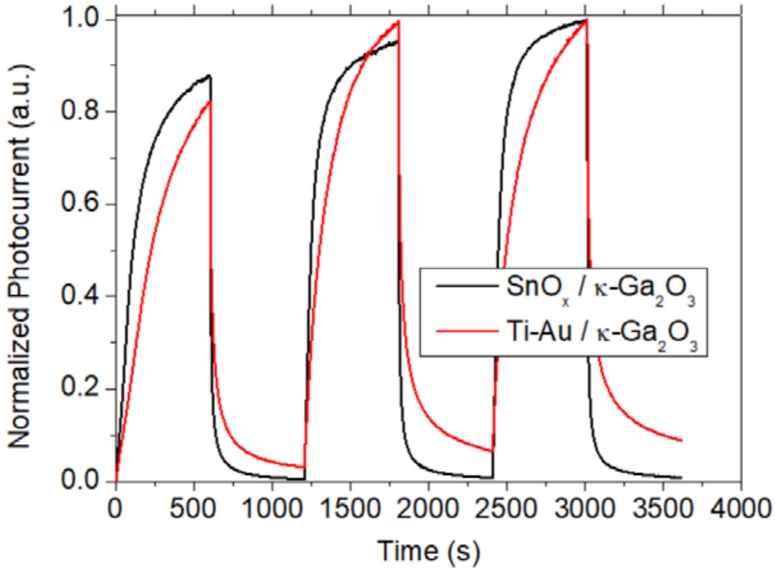

Figure S2. Comparison between the photoresponse in time of photoresistors with SnO_x and Ti/Au contacts. Three cycles of on-off illumination with photons at 254 nm were acquired in the region at high voltages (200 V) for a distance between adjacent contacts of 200 μm (pair 1–2). The irradiance is set at 50 μW/cm^2.
